# Prenatal cadmium exposure is associated with shorter leukocyte telomere length in Chinese newborns

**DOI:** 10.1186/s12916-019-1262-4

**Published:** 2019-02-06

**Authors:** Lina Zhang, Lulu Song, Bingqing Liu, Mingyang Wu, Lulin Wang, Bin Zhang, Chao Xiong, Wei Xia, Yuanyuan Li, Zhongqiang Cao, Youjie Wang, Shunqing Xu

**Affiliations:** 1 Department of Maternal and Child Health, School of Public Health, Tongji Medical College, Huazhong University of Science and Technology, Hangkong Road 13, Wuhan, 430030 Hubei China; 2 Wuhan Children’s Hospital (Wuhan Maternal and Child Healthcare Hospital), Tongji Medical College, Huazhong University of Science and Technology, Wuhan, Hubei China; 3 Key Laboratory of Environment and Health, Ministry of Education and Ministry of Environmental Protection, and State Key Laboratory of Environmental Health (Incubation), School of Public Health, Tongji Medical College, Huazhong University of Science and Technology, Hangkong Road 13, Wuhan, 430030 Hubei China

**Keywords:** Prenatal urinary cadmium, Telomere length, Cord blood, Newborns

## Abstract

**Background:**

Newborn telomere length (TL) is considered a potential marker for future disease and lifelong health, but few epidemiological studies have examined the determinants of TL in early life. The study aim was to investigate whether there is an association between prenatal cadmium exposure and relative cord blood TL in Chinese newborns.

**Methods:**

Participants were 410 mother–newborn pairs drawn from a prospective birth cohort study conducted in Wuhan, China, between November 2013 and March 2015. Urine samples were collected from pregnant women during their period of institutional delivery. Urinary cadmium concentrations were measured by inductively coupled plasma mass spectrometry. The real-time quantitative polymerase chain reaction detection was used to measure relative TL using genomic DNA isolated from umbilical cord blood leukocytes. Multivariate linear regression models were used to estimate the effect of prenatal urinary cadmium concentration on relative cord blood TL.

**Results:**

The geometric mean of maternal urinary cadmium concentration was 0.68 μg/g creatinine. In the multivariate-adjusted linear regression model, per doubling of maternal urinary cadmium concentration was associated with 6.83% (95% CI − 11.44%, − 1.97%; *P* = 0.006) shorter relative cord blood TL. Stratified analyses indicated that the inverse association between prenatal urinary cadmium and newborn relative TL was more pronounced among female infants and mothers < 29 years, while there were no significant effect modification according to infant sex (*P* for interaction = 0.907) and maternal age (*P* for interaction = 0.797).

**Conclusions:**

The findings indicated that increased maternal urinary cadmium was associated with shortened relative cord blood TL. The results provide more evidence of the negative effects of environmental cadmium exposure and suggest that accelerated aging or cadmium-related diseases may begin in early life.

## Background

Cadmium is a heavy metal that is toxic at low levels. Cadmium is ubiquitously present in the environment mainly owing to human activities, such as mining, coal burning, industrial emissions, household wastes, and the using and disposing of cadmium-containing products [1]. Consumption of food containing cadmium and cigarette smoking are two major causes of cadmium exposure [2]. In addition, contaminated dust and air can contribute to cadmium exposure [3]. Once absorbed, cadmium efficiently accumulates in the human body, mainly in the kidneys, where it is retained with a biological half-time around 10–30 years [2]. Urinary cadmium concentration mainly influenced by the body burden of cadmium is proportional to the concentration in the kidney [2, 3]. Women, particularly pregnant women, accumulate more cadmium than men [4, 5]. Epidemiological studies have reported that prenatal cadmium exposure increased the risk of adverse perinatal outcomes, such as preterm birth [6, 7] and lower birth weight [8–10].

Telomeres are nucleoprotein structures located at the end of chromosomes; their function is to maintain chromosome integrity and genome stability [11]. Telomere shortening occurs during each DNA replication cycle in somatic cells, as DNA polymerase cannot completely replicate the end telomeric DNA during cellular division [12]. Shortened telomere length (TL) is associated with aging-related diseases, such as type 2 diabetes mellitus [13, 14], cardiovascular disease [15, 16], and cancer [17, 18].

TL variation in later life is determined by the initial or newborn setting of TL and the subsequent attrition rate [19]. TL at birth is an important predictor for TL in adulthood [20]. A small but growing body of epidemiological studies have explored the relationship between newborn TL and prenatal environmental exposure, such as perfluoroalkyl and polyfluoroalkyl [21], intrauterine tobacco exposure [22], polycyclic aromatic hydrocarbons [23], residential traffic exposure [24], and residential fine particulate matter (PM 2.5) [25]. One cross-sectional study, conducted by Lin et al, found that cadmium concentration in placental tissue was negatively related to placental TL among Chinese participants living in metal polluted areas [26]. No study to date has evaluated whether prenatal cadmium exposure is associated with the alteration of newborn leukocyte TL in the general population. As cadmium can cross the placental barrier, maternal cadmium is strongly associated with cadmium concentrations in newborn umbilical cord blood [27, 28]. We hypothesized that prenatal cadmium exposure might affect newborn TL. Here, we intended to evaluate the association between maternal urinary cadmium concentration, a marker of long-time cumulative exposure, and relative cord blood TL in a Chinese population and to provide evidence that shorter TL is a possible mechanism of cadmium-induced toxicity.

## Methods

### Study population

The study was conducted from November 2013 to March 2015 at the Wuhan Children’s Hospital (Wuhan Maternal and Child Healthcare Hospital), a tertiary medical institution in Wuhan city, China. A total of 418 mothers met the following enrollment criteria: (1) lived in Wuhan city, Hubei province (in central China), (2) with a singleton gestation, (3) had received prenatal care and intended to deliver at the study hospital, and (4) agreed to provide a urine sample and fill out a questionnaire. Eight newborns had poor quality cord blood DNA; therefore, 410 mother–newborn pairs were included in the final analysis. The study protocol was approved by the ethics committee of Tongji Medical College, Huazhong University of Science and Technology, and the Wuhan Children’s Hospital (Wuhan Maternal and Child Healthcare Hospital). Individual written informed consent was obtained from all mothers at enrollment.

### Urine sample collection and cadmium measurement

Maternal spot urine samples were collected in the study hospital during the period of mothers waiting for labor (within 3 days before delivery), and all urine samples were stored at − 20 °C in polypropylene tubes before measurement.

Urinary metal levels were measured using inductively coupled plasma mass spectrometry (ICP-MS) (Agilent 7700; Agilent Technologies). In brief, frozen urine samples were thawed at room temperature (20 °C–25 °C), then each urine sample was mixed thoroughly using a vortex mixer, and 1.2% (*v*/*v*) HNO3 was introduced for overnight nitrification, and then the sample was digested at 40 °C for 1 h. Cadmium concentration measurement was conducted for each sample using ICP-MS. A control urine sample of standard reference material (SRM2670a Toxic Elements in Urine; National Institute of Standards and Technology, Gaithersburg, MD, USA) was used for external quality control in each batch. The low and high certified concentrations (μg/L) of cadmium were 0.059 ± 0.0034 and 5.16 ± 0.11, respectively. All concentration measurements were within the certified range (5%). Interday and intraday variations of cadmium were tested using cadmium concentrations measured from the standard reference material human urine sample. The interday and intraday coefficients of variation (CVs) were 0.36% and 0.89%, respectively. The limit of detection for cadmium was 0.001 μg/L. There were no undetected samples in the present study.

Urinary creatinine was measured using a creatinine kit (Mindray BS-200 CREA Kit; Shenzhen Mindray Bio-medical Electronics Co., Ltd). Creatinine-corrected urinary cadmium concentrations (μg/g creatinine) were used in the final statistical analysis and to account for the effect of variation in spot urine diluteness.

### Cord blood collection and relative leukocyte TL examination

Umbilical cord blood samples from newborns were collected immediately postpartum. Plasma and blood cells were separated and stored at − 80 °C for later analysis.

Genomic DNA in each cord blood sample was isolated using a Wizard® Genomic DNA Purification Kit (Promega Corporation, Madison, WI, USA). DNA samples with an A260/A280 ratio within the range 1.8 to 2.0 were considered eligible. Relative TL in each DNA sample was examined using the real-time quantitative polymerase chain reaction (qPCR) method. Relative TL was determined using the ratio of telomere repeat copy number to single-copy gene copy number (T/S ratio). Two primer pairs were used for the amplification of telomeric gene and single-copy gene (human β-globulin (HBG)), respectively. The primer sequences of telomeric gene and single-copy gene (HBG) were as follows: Forward primer (Tel-Forward): 5′-ACACTAAGGTTTGGGTTTGGGTTTGGGTTTGGGTTAGTGT-3′; reverse primer (Tel-Reverse): 5′-TGTTAGGTATCCCTATCCCTATCCCTATCCCTATCCCTAACA-3′; forward primer (HBG-1): 5′-GTGCACCTGACTCCTGAGGAGA-3′; reverse primer (HGB-2): 5′-CCTTGATACCAACCTGCCCAG-3′. For telomeric gene, the reaction mixture contained 10 ng genomic DNA, 1× KAPA SYBR® FAST qPCR kit Master MIX (KAPA Biosystems), 270 nM of the forward, and 900 nM of the reverse; RNase-free water was added to a final volume of 10 μL. For single-copy gene (HBG), the reaction mixture contained 10 ng genomic DNA, 1× SYBR FAST qPCR kit Master MIX (KAPA Biosystems), 200 nM of the forward, and 200 nM of the reverse; RNase-free water was added to a final volume of 10 μL. Each sample was assayed in triplicate in a 384-well plate using the ViiA™ 7 Dx Real-Time PCR System (Applied Biosystems). The standard deviation (SD) of the Ct values of three replicates for each sample below 0.30 was considered a repeatable result; otherwise the measurement was conducted again in a new 384-well plate. The PCR cycling conditions were 50 °C for 2 min and 95 °C for 3 min to activate DNA polymerase, then 40 cycles of denaturation at 95 °C for 3 s and annealed/extended at 60 °C for 30 s. Melting curve analysis was included at the end of each run to validate the specificity of the PCR products. Standard curve, plotted using a standard reference genomic DNA sample, is a 5-point curve produced using 1:4 serial dilutions of DNA concentration from 104 to 0.4 ng/μL (*R*^*2*^ ≥ 0.99). The standard reference genomic DNA sample was derived from a mixture of genomic DNA of 50 samples randomly selected from our study population. On each run, a 5-point serial dilution of standard reference genomic DNA sample was run to assess qPCR amplification efficiency. The qPCR amplification efficiency in the present study was 108% for telomeres and 103% for single-copy gene. To examine the interrun reproducibility, we set three replicates for standard reference genomic DNA sample in each 384-well plate, and the CV for interrun was calculated using Ct values of single-copy gene of standard reference sample in all qPCR assays. The interrun and intrarun CVs of single-copy gene were 4.1% and 3.0%, respectively. The CVs within triplicates of the telomere runs and single-copy gene runs were 0.53% and 0.74%, respectively.

### Assessment of covariates

Information on maternal age at delivery (in years), education, and active/passive smoking during pregnancy was obtained via standard questionnaires administered by trained nurses at the study hospital during the period of institutional delivery. Information on infant sex, gestational age (in weeks) at birth, parity, gestational diabetes mellitus, and hypertensive disorders in pregnancy were obtained from medical records. Passive smoking refers to second-hand smoking exposure during pregnancy. The self-reported date of last menstrual period was used to estimate gestational age. Maternal prepregnancy weight was self-reported and height was measured using a stadiometer at the first prenatal care visit in the study hospital, generally during the first trimester of pregnancy. Prepregnancy body mass index (BMI) (kg/m^2^) was expressed as the ratio of weight in kilograms (kg) and the square of height in meters (m^2^). Hypertensive disorders in pregnancy in this study included chronic hypertension, pre-eclampsia, and gestational hypertension.

### Statistical analysis

All database managements and statistical analyses were performed using SAS 9.4 software (SAS Institute Inc., Cary, NC, USA). The Shapiro–Wilk test was used to test the distribution of continuous data. Population characteristics were described as mean ± SD for normally distributed data, median (interquartile range, IQR) for skewed data, and numbers (percentages) for categorical data. Relative cord blood TL and metal concentrations were natural log (Ln)-transformed to approximate a normal distribution.

We used a multivariate linear regression model to estimate the regression coefficient (*β*) and standard error (SE) of the association between urinary cadmium concentration (Ln-transformed) and relative cord blood TL (Ln-transformed). The percentage change in relative TL for per doubling of urinary cadmium concentration was estimated as (*e*^(ln2 × *β*)^− 1) × 100%, and the 95% confidence interval (95% CI) was estimated as (*e*^[ ln 2 × (*β* ± 1.96 × SE]^− 1) × 100%. To ensure the robustness of the results, sensitivity analyses were performed omitting subjects with gestational diabetes mellitus and hypertensive disorders in pregnancy. To evaluate potential modification effects, further analyses were sub-grouped by infant sex (male or female) and maternal age at delivery (< 29 or ≥ 29 years, the median value). The Wald test was used to test the interaction across subgroups.

Covariates allowed in the regression model were selected based on published literatures [22, 29–32] or if they individually caused > 10% of the changes in the effect estimates [33]. The covariates in the adjusted models were infant sex (male or female), gestational age (continuous, weeks), maternal age at delivery (continuous, years), education (junior school or below, high school, college or above), prepregnancy BMI (continuous, kg/m^2^), prenatal passive smoking (yes or no), parity (primiparous or multiparous), hypertensive disorders in pregnancy (yes or no), gestational diabetes mellitus (yes or no), urinary zinc (Ln-transformed, μg/g creatinine), and urinary selenium (Ln-transformed, μg/g creatinine). We did not adjust for maternal smoking because all mothers in this study were non-smokers. There were no missing data for the covariates added in the adjusted models.

## Results

The characteristics of the mother–newborn pairs are shown in Table 1. Of the newborns, 206 (50.2%) were males and 204 (49.8%) were females. The mean ± SD gestational age was (39.4 ± 1.2) weeks. The participating mothers were 22 to 44 years old, with an average age of 29.2 ± 3.4 years. Of the 410 mothers, 313 (76.4%) had an educational level of college or above, 133 (32.4%) were exposed to environmental tobacco smoke during pregnancy, and 339 (82.7%) were primiparas.

**Table 1:** Characteristics of the 410 mother–newborn pairs in present study

Variables	Mean ± SD/ Median (IQR)/ Number (%)
Infant sex	
Male	206 (50.2)
Female	204 (49.8)
Gestational age (weeks)	39.4 ± 1.2
Maternal age at delivery(years)	29.2 ± 3.4
Education	
Junior school or below	28 (6.8)
High school	69 (16.8)
College or above	313 (76.4)
Passive smoking during pregnancy	
Yes	133 (32.4)
No	277 (67.6)
Prepregnancy BMI (kg/m^2^)	21.2 ± 3.0
Parity	
Primiparous	339 (82.7)
Multiparous	71 (17.3)
Hypertensive disorders in pregnancy	
Yes	12 (2.9)
No	398 (97.1)
Gestational diabetes mellitus	
Yes	41 (10.0)
No	369 (90.0)
Relative cord blood TL	0.74 (0.56–0.92)

Data presented as mean ± SD for normally distributed data, or median (IQR) for skewed distributed data, or numbers (percentage) for categorical data

Table 2 shows the maternal urinary cadmium concentrations. The geometric means of the uncorrected and creatinine-corrected urinary cadmium concentrations were 0.33 μg/L (5th–95th percentile, 0.09–1.16 μg/L) and 0.68 μg/g creatinine (5th–95th percentile, 0.30–1.84 μg/g creatinine), respectively.

**Table 2:** Distribution of maternal urinary cadmium concentrations

	Geometric mean	Mean ± SD	Percentiles				
			5th	25th	50th	75th	95th
Uncorrected cadmium (μg/L)	0.33	0.44 ± 0.35	0.09	0.19	0.35	0.59	1.16
Creatinine-corrected cadmium (μg/g creatinine)	0.68	0.82 ± 0.70	0.30	0.47	0.66	0.96	1.84

Table 3 shows that prenatal urinary cadmium was significantly and inversely associated with relative cord blood TL in the unadjusted and adjusted models. In the unadjusted model, per doubling of urinary cadmium contributed to 5.30% (95% CI − 9.63%, − 0.77%; *P* = 0.022) shorter relative cord blood TL. After adjustment for newborn characteristics (infant sex and gestational age), a doubling of urinary cadmium was associated with 5.34% (95% CI − 9.66%, − 0.81%; *P* = 0.022) shorter relative cord blood TL. After further adjustment for maternal characteristics (maternal age at delivery, education, passive smoking during pregnancy, prepregnancy BMI, parity, hypertensive disorders in pregnancy and gestational diabetes mellitus), prenatal urinary cadmium remained significantly and inversely associated with relative TL; a doubling of urinary cadmium was associated with 5.73% (95% CI − 10.18%, − 1.06%; *P* = 0.017) shorter relative cord blood TL. After further adjustment for urinary zinc and urinary selenium, per doubling of maternal urinary cadmium contributed to 6.83% (95% CI: − 11.44%, − 1.97%; *P* = 0.006) shorter relative cord blood TL.

**Table 3:** Percentage change in relative TL by prenatal cadmium exposure (*N* = 410)

	Percentage change(%)* (95% CI)	*P* value
Unadjusted	− 5.30 (− 9.63, − 0.77)	0.022
Model A	− 5.34 (− 9.66, − 0.81)	0.022
Model B	− 5.73 (− 10.18, − 1.06)	0.017
Model C	− 6.83 (− 11.44, − 1.97)	0.006

*Estimates are percentage changes in relative cord blood TL of per doubling of urinary cadmium concentration

Model A, adjustment for newborn characteristics (infant sex and gestational age)

Model B, additionally adjustment for maternal characteristics (maternal age at delivery, educational level, passive smoking in pregnancy, prepregnancy BMI, parity, hypertensive disorders in pregnancy, and gestational diabetes mellitus)

Model C, additionally adjustment for zinc (Ln-transformed, μg/g creatinine) and selenium (Ln-transformed, μg/g creatinine)

In sensitivity analyses (Table 4), the results were essentially unchanged after excluding data for 12 mothers with hypertensive disorders in pregnancy and 41 mothers with gestational diabetes mellitus.

**Table 4:** Sensitivity analyses

	*n*	Percentage change(%)* (95% CI)	*P* value
Overall	410	− 6.83 (− 11.44, − 1.97)	0.006
Excluding hypertension disorders in pregnancy	398	− 6.92 (− 11.60, − 1.98)	0.006
Excluding gestational diabetes mellitus	369	− 6.54 (− 11.52, − 1.28)	0.015

Models’ adjustments are according to model C in Table 3. *Estimates are percentage changes of relative cord blood TL of per doubling of urinary cadmium concentration

Table 5 shows the results of the subgroup analyses. In the fully adjusted model, percentage changes in relative cord blood TL of per doubling of maternal urinary cadmium were − 5.52% (95% CI − 12.24%, 1.71%) in males, − 8.26% (95% CI − 14.45%, − 1.63%) in females, − 8.47% (95% CI − 15.45%, − 0.91%) in mothers < 29 years, and − 5.29% (95% CI − 11.28%, 1.11%) in mothers ≥ 29 years. No significant interactions were found between prenatal urinary cadmium and infant sex (*P* for interaction = 0.907) or maternal age (*P* for interaction = 0.797) regarding the alteration of relative cord blood TL.

**Table 5:** Associations between prenatal cadmium exposure and relative cord blood TL, stratified by infant sex and maternal age

Subgroup	*n*	Percentage change(%)^*^ (95% CI)	*P* for interaction
Infant sex			0.907
Male	206	− 5.52 (− 12.24, 1.71)	
Female	204	− 8.26 (− 14.45, − 1.63)	
Maternal age at delivery (years)			0.797
< 29	201	− 8.47 (−15.45, − 0.91)	
≥ 29	209	− 5.29 (−11.28, 1.11)	

Models’ adjustments are according to model C in Table 3. *Estimates are percentage changes in relative cord blood TL of per doubling in urinary cadmium concentration

## Discussion

The present results indicate that prenatal cadmium exposure was inversely associated with relative cord blood TL after adjustment for potential confounders; per doubling of prenatal urinary cadmium contribute to 6.83% shorter relative cord blood TL.

Maternal urinary cadmium measured during delivery was a useful indicator of maternal environmental cumulative cadmium exposure throughout pregnancy or over a longer period. In the present study, cadmium was detected in all maternal urine samples, indicating that participating mothers were widely exposed to cadmium in their daily lives. Maternal urinary cadmium concentrations in the present study (geometric mean 0.33 μg/L or 0.68 μg/g creatinine; median 0.35 μg/L or 0.66 μg/g creatinine) were higher than those reported in the USA (geometric mean 0.31 μg/g creatinine) [9] and on average comparable to those found in Europe (median 0.31 μg/L) [34] and Japan (geometric mean 0.77 μg/g creatinine) [35], but lower than those reported in Bangladesh (median 0.63 μg/L) [10].

Our detection of shorter relative TL associated with increased cadmium exposure is consistent with findings from three previous studies. One study [26] revealed that cadmium concentration in placental tissue was negatively correlated with placental TL among Chinese participants in areas known to be polluted by metals. However, placental tissue is usually contaminated by maternal tissue or blood and so may not be suitable for measuring newborn TL. The other two studies conducted on adults [36] and adolescents [37] demonstrated that higher levels of cadmium exposure were associated with shorter leukocyte TL. Only one previous study [38] conducted on 8-year-old children in Poland near a site of industrial pollution, reported a null association between cadmium exposure and telomere shortening, possibly because of the relatively small sample size (*n* = 99).

There are some potential biological mechanisms underlying the association between prenatal cadmium exposure and shortened relative cord blood TL. Both experimental [39] and epidemiological studies [40–42] have reported a positive association between environmental cadmium exposure and oxidative stress. Telomeres are sensitive to oxidative damage because of their high guanine content [43]. Reactive oxygen species, particularly hydroxyl radicals, can result in single-strand breaks, and telomeric DNA may be deficient in repairing single-strand breaks compared with genomic DNA [44]. Thus, oxidative stress may be a mechanism by which cadmium exposure leads to telomere shortening. Furthermore, chronic cadmium exposure can stimulate the cumulative load of inflammatory cytokines [42, 45, 46], including TNF-a, IL-6, C-reactive protein, and fibrinogen. Elevated levels of inflammation can contribute to telomere shortening by accelerating cell turnover and cell replicative senescence, as well as by induction of oxidative stress and regulating telomerase activity [47]. In addition, cadmium is a potent carcinogen and can disturb human DNA repair systems, particularly the excision and mismatch repair systems [48]. Whether these processes affect telomere maintenance in fetuses requires further research.

The sex-specific effect of maternal cadmium exposure on neonatal adverse outcomes has been documented, with increased susceptibility of females [10, 49, 50]. In the stratified analysis, we found that the inverse association between maternal cadmium exposure and relative cord blood TL was more pronounced in female infants. The underlying mechanisms are unclear. One possible explanation is that the sex difference in cadmium-induced changes in epigenetic regulation of genes governing telomere dynamics. Studies have reported that cadmium exposure in early life caused different DNA methylation pattern in girls and boys [51]. Kipple et al. found that methylated sites for genes involved in cell death were sex different [52]. Cell death-related genes were important regulators for telomere attrition rate [53]. Another possible explanation is the action of insulin-like growth factor 1 (IGF-1). Epidemiological studies demonstrated that lower level of IGF-1 was associated with shorter TL [54, 55]. Cadmium exposure was found to be associated with lower concentration of IGF-1 [56]. We speculated that female newborns, who was reported to have higher level of IGF-1 in cord blood/plasma than males [57, 58] might suffer more cadmium-induced telomere shortening. The stratified analysis also indicated that the inverse association between prenatal cadmium exposure and newborn relative TL was more evident among mothers < 29 years. Telomere attrition was an important indicator of aging [59]. A reasonable explanation might be that the risk factors for biological aging might mask the effect of maternal cadmium exposure on newborn TL among high-risk mothers (≥ 29 years) and leave adverse effect to be manifested among relatively healthy mothers (< 29 years).

TL in adults is associated with age-related diseases and mortality [13–18]. The initial or newborn setting of TL is a major determinant of TL in later life [19, 20]. Our findings provide additional evidence that diseases in later life may have their roots in early life. Furthermore, cadmium exposure is associated with signs of aging, such as mitochondrial dysfunction [60], genomic instability [61], and cell senescence [62]. Considering that telomere attrition is an important biomarker of aging [59], our results add to the accumulating evidence on the adverse effects of cadmium exposure on aging.

The potential limitations of this study should be noted. First, we did not measure cord blood cadmium concentrations, which might provide direct measure of fetal cadmium exposure. Researches showed that maternal cadmium level is highly associated with cadmium concentrations in newborn cord blood [27, 28]. Second, spot urinary cadmium concentration might not precisely reflect cadmium exposure throughout pregnancy. However, previous studies have shown that urinary cadmium levels are rather constant over the pregnancy period [63] and are stable at multiple points across a day [64]. As the half-time of cadmium in the human body is very long, urinary cadmium concentration is considered a better surrogate for cumulative exposure [2, 65, 66]. Third, although our results were consistent after multiple adjustments, additional unmeasured or unknown confounding factors, such as cord blood cell type, newborn telomerase activity, and maternal cotinine level may have biased our findings. Finally, our sample was drawn from a Chinese population, so caution is needed before generalizing the results to other populations.

## Conclusions

In summary, prenatal exposure to cadmium was associated with decreased newborn cord blood TL. The results provide more evidence of the negative effects of environmental cadmium exposure and suggest that accelerated aging or cadmium-related diseases may begin in early life.

## Abbreviations

BMI: Body mass index
CI: Confidence interval
HBG: Human β-globulin
ICP-MS: Inductively coupled plasma mass spectrometry
IGF: Insulin-like growth factor
IQR: Interquartile range
Ln: Natural log
qPCR: Real-time quantitative polymerase chain reaction
SD: Standard deviation
TL: Telomere length
